# Relation and Treatment Approach of Migraine in Pregnancy and Breastfeeding

**DOI:** 10.7759/cureus.36828

**Published:** 2023-03-28

**Authors:** Tileshkumar Turankar, Akshayata Sorte, Mayur B Wanjari, Swarupa Chakole, Shantanu Sawale

**Affiliations:** 1 Community Medicine, Jawaharlal Nehru Medical College, Datta Meghe Institute of Medical Sciences, Wardha, IND; 2 Medicine, Jawaharlal Nehru Medical College, Datta Meghe Institute of Medical Sciences, Wardha, IND; 3 Department of Research and Development, Jawaharlal Nehru Medical College, Datta Meghe Institute of Higher Education and Research, Wardha, IND; 4 Community Medicine, Datta Meghe Institute of Medical Sciences, Wardha, IND

**Keywords:** nsaids, acetaminophen, non-pharmacological treatment, preconception counseling, lactation, pregnancy

## Abstract

Headaches are one of the most frequent reasons people visit the neurology department. In 2019, headache issues ranked as the 14th most common cause of disability-adjusted life years globally. According to the International Headache Society, migraine is a particular type of headache that is unilateral, frequently throbbing, associated with vertigo, and sensitive to light, sound, and head movement. A migraine has four stages: premonitory, aura, headache, and postdrome. Migraine is the type of discomfort that most frequently complicates the pregnancy. A migraine is more common in women than in men. Migraines are influenced by increased levels of estrogen during pregnancy and a sharp decrease in those levels during puerperium. Untreated migraine can result in premature labor, hypertension, and low birth weight babies. Menstrual-related events occurred more frequently in migraine sufferers than in non-migraine sufferers. We have explained the relation of sex hormones that trigger migraine. We have also reviewed the therapeutic approach, such as pharmacological and non-pharmacological approaches, for migraine in pregnancy and breastfeeding. A migraine episode during menstruation was slightly more severe and complicated than a headache that wasn't a migraine. Breastfeeding is not prohibited by migraines. The steady estrogen levels brought on by lactating women's lack of menstruation may function as a protective factor. In addition to any required drug therapy, nonpharmacological techniques should always be used as the first line of treatment. Preconception counseling is an essential part of providing headache patients with safe therapy during pregnancy. Supplemental estrogen should not be used by any women who have an aura or who are 35 years of age or older because there is inadequate proof to support any long-term adverse effects. Paracetamol is advised for use in acute therapy during pregnancy. Mothers who used acetaminophen during pregnancy are more likely to have children with hyperkinetic disorders and characteristics resembling attention-deficit/hyperactivity disorder. Menstrual migraine can be treated in a variety of ways, including acute therapy, non-pharmacological therapy, and preconception counseling. Similar tactics are used to treat migraines during pregnancy, but it's important to take the medications' safety rating into account. Migraines and menstrual problems go together. A safeguarding element is a constant estrogen level during pregnancy and breastfeeding. The preferred method of treatment for migraine is non-pharmacological therapy, followed by prenatal counseling. Sumatriptan and acetaminophen are both effective treatments for transient migraine attacks that occur during pregnancy or breastfeeding.

## Introduction and background

One of the most popular causes of consultations with neurology sections is headaches [1]. Headache problems were the 14th leading source of disability-adjusted life years worldwide in 2019 [2]. According to the Global Burden Disease statistics, migraines account for 47.2 million in terms of years lived with disabilities (YLDs) (95% CI: 30.0-68.7), placing them second among all other sources of disability globally in terms of YLDs. A worldwide all-age point prevalence of 18% was calculated based on an expected 1.3 billion (95% CI: 1.2-1.4) affected individuals [3]. The International Headache Society defines migraine as a headache condition with specific characteristics, such as being unilateral and often throbbing, as well as being linked with nausea and sensitivity to light, sound, and head movement [4]. Genetic and environmental variables are likely to be the root causes of migraine initiation, recurrence, and relief [5]. The prevalence of migraine is seen more in females than in males [6]. The pain condition that most frequently makes pregnancy more complicated is migraine. Primary headaches (migraine, tension, cluster, and other trigeminal sympathetic cephalgias) are widespread during pregnancy and after childbirth [7,8]. According to several studies, between 50% and 75% of pregnant women who suffer from migraines see a decrease in the incidence or complete cessation of their episodes mainly in the second and third trimesters [9]. Important "red flags" to take into account during pregnancy include hypertension, aberrant neurological symptoms, and new or refractory pain. The prevalence of migraine in females is 17.1% and in males is 5.6% for the duration of one year [10,4]. Elevated amounts of estrogen during pregnancy and a sudden drop in those levels during puerperium have an impact on migraines. Migraine that goes untreated can cause preterm delivery, preeclampsia, and infants with low birth weights [10,11]. According to Italian research, during pregnancy, 9% of females suffering from headache change the pattern of headaches from tension-type headaches to migraines without aura [12]. Migraine is associated with menstruation. According to one research, women with migraines experienced menstruation-related episodes more frequently than women without migraines (67.7% versus 29.5%). The severity and complexity of a migraine attack during menstruation were marginally higher than those of a non-migraine headache attack [13]. Migraine is likely to persist throughout pregnancy and the following period if it does not get better by the end of the first trimester [14]. First-time migraine during pregnancy is more prone to have an aura [15]. Not just migraineurs experience postpartum headaches; about 30-40% of all women do [16]. This review helps in improving the knowledge of migraine during pregnancy and breastfeeding and provide treatment modalities for migraine.

## Review

Methodology

We reviewed PubMed, Scopus, and the Web of Science database for articles on migraine during pregnancy and breastfeeding. We searched for and included as many relevant studies as possible using the medical subject heading phrases "pregnancy" and "migraine disorder," as well as various keyword combos like lactation, estrogen, menstrual migraine, and migraine attack. To identify potential additional records from other sources, a literature search was conducted to locate case-control studies and potential earlier reviews and meta-analyses.

Phases of migraine

Knowing the four stages of migraine, which include premonitory, aura, headache, and postdrome, is the first step in knowing how migraines relate to pregnancy or how to manage them. The timeline and symptoms related to each period are listed in Table 1.

**Table 1:** Representing the phases of migraine and associated symptoms and timeline

Phase	Associated symptom	Timeline
Premonitory	Fatigue, cognitive difficulties, food cravings, neck pain, yawning	Few hours to days
Aura	Scotoma, speech disturbance, motor symptoms	5 to 60 minutes
Headache	Headache associated with nausea or without vomiting, photophobia, phonophobia	6 to 80 hours
Postdrome	Feeling tired, difficulty with concentration, neck stiffness	Up to 24 hours

Relationship between migraine and hormones

Some studies showed that the menstrual period and migraine are related. According to one research, migraines can be brought on by estrogen cessation [17,18]. Menstruation is a significant menstruation migraine cause [19]. When a woman is menstruating, her migraine attacks are more intense, incapacitating, and common than when she is mid-luteal or mid-cycle. Menstruation and migraine without aura were clearly linked, but migraine with aura (MwA) was not. According to research, conditions of excessive estrogen and estrogen abstinence both cause MwA [20]. Compared to women who experience migraine without aura, women who experience migraine with aura have higher circulating estradiol levels [21]. Progesterone metabolites may help control migraine attacks that occur during the luteal phases of the menstrual period [22]. It is well known that feminine sex hormones play a part in the pathogenesis of migraine. In exogenous estrogen users, the decline in estrogen levels at the end of the menstrual cycle and the start of the hormone-free period is a key factor in the pathogenesis of headaches in women [23]. Migraine is associated with genes as well as the menstrual period. According to one research, estrogen receptor 1 (ESR1) and estrogen receptor 2 (ESR2) may contribute to the hereditary basis of migraine [24].

Relationship between migraine and pregnancy and breastfeeding

It has long been thought that migraines could be a sign of pregnancy problems [11]. There is a strong correlation between pregnancy and migraine, with research indicating that 80% of women who have previously had migraine can experience them while pregnant [25]. A large release of prostaglandins during the menstrual period of the cycle might be able to account for the greater incidence of dysmenorrhea among patients with migraine without aura. The function of these substances in causing dysmenorrhea has long been known, and it has been hypothesized that women with menstrual migraine have unusually elevated plasma prostaglandin levels during this time [26]. Migraine does not preclude breastfeeding [27]. In both the first week and the first month after delivery, breastfeeding was beneficial and reduced the recurrence of migraine return [10,16]. It is possible that the stable estrogen levels due to the absence of menstruation during lactation may have a protective effect [28]. Using sumatriptan for migraine while breastfeeding is also harmless [29].

Treatment for migraine

According to one research, women with migraine without aura have a higher chance of recovering than those with migraine with aura [30,31]. However, the most ignored area of migraine is early diagnosis, follow-up, and non-hormonal symptomatic and preventive treatments [32]. Nonpharmacological methods should always be used as the first line of treatment in addition to any necessary medication therapy [27,33,34]. Preconception counseling is a crucial component of giving patients with headaches who are childbearing potential secure treatment [9,35]. As certain medications carry a danger to the fetus, pregnancy and breastfeeding can make it more difficult for women with migraine to receive the right therapy. Therefore, it is essential that patients must be given the knowledge necessary to make an educated decision regarding the use of medicines during pregnancy, including a talk of the risk-benefit ratio. A smaller trial of 18 patients found a 41% decrease in the total frequency of headaches, and an earlier retrospective analysis of 1,300 women found a 67% cessation of migraine in pregnancy [11]. There are several documented causes of migraine, including stress and hormonal shifts [36]. Hormonal treatments are linked to a shift in migraine pattern that is commonly seen during pregnancy, puerperium, puberty, and after menopause [30,37]. However, one study concluded that the majority of women suffering from migraine do not use hormone therapy as their first choice of treatment. All women with aura and those who are 35 years of age or later should not use supplemental estrogen because there is insufficient evidence to support its long-term negative effects [32]. The recommended medication for acute therapy during pregnancy is paracetamol. One study mentioned that maternal hormones play a crucial role in controlling embryonic brain development. The use of paracetamol or acetaminophen can interfere with maternal hormones or via neurotoxicity by inducing oxidative stress, which can lead to the loss of neurons [38]. Furthermore, one study revealed that mothers of children who experience hyperkinetic disorder (HKD) and traits similar to ADHD are more likely to use paracetamol during pregnancy [38]. Sumatriptan use on occasion can be explored if paracetamol is insufficiently efficient. Ibuprofen and other nonsteroidal anti-inflammatory medications (NSAIDs) can also be used in certain situations, but doing so during the first and third trimesters carries certain risks and is not advised [39]. The cornerstones of acute migraine treatment are NSAIDs and triptans. Ibuprofen and naproxen are NSAIDs that can be used as a second choice, but they shouldn't be used for extended amounts of time and should be avoided in the third trimester [34]. Behavioral strategies, paracetamol/acetaminophen, NSAIDs, opioids/barbiturates, triptans, dihydroergotamine, and nerve blocks are among the common acute therapies for pregnant women [10]. A dosage of aspirin of less than 100 mg seems to be harmless. Due to the danger of postpartum and neonatal bleeding, as well as premature ductus arteriosus closing, higher dosages should be avoided in the third trimester [35]. For individuals experiencing episodes of slight to intermediate severity, the acetaminophen-NSAID combination is appropriate. Patients with severe attacks and those with moderately severe attacks who do not react well to NSAIDs should consider the triptan approach [40]. One study revealed that chlorpromazine, dimenhydrinate, and diphenhydramine can be used to manage severe migraine episodes [34]. NSAIDs and acetylsalicylic acid are two popular over-the-counter painkillers used by many migraine sufferers. These should not be used in the third trimester in part due to the higher chance of bleeding and premature ductus arteriosus closing [33]. Meperidine and morphine have no danger in humans, but they shouldn't be used at the end of the third trimester, according to the risk classifications set by the US FDA. Dexamethasone or prednisone may be attempted in some refractory instances. Six hundred fifty-eight Swedish women who were exposed to sumatriptan in the first trimester of pregnancy did not have an increased teratogenic risk, according to an analysis of the Swedish birth registry [41]. There were no variations in teratogenicity, the rate of abortion, or the number of premature deliveries between pregnant women taking triptans and those who did not, according to a meta-analysis of over 4,000 women [42]. An observational survey conducted recently supported these conclusions [43]. Metoclopramide and diphenhydramine together may be a more efficient therapy for migraine or tension headaches than codeine [33]. Triptans might not have any negative impacts on a fetus or child. Low-dose aspirin may not be linked to fetal/child deleterious effects, but acetaminophen, prednisolone, indomethacin, ondansetron, antipsychotics, and parenteral magnesium may be. The danger of addiction and the availability of more effective medications make opioid use for migraines generally avoided. Prolonged use of opioids is linked to respiratory depression, growth stunting, and newborn withdrawal in later pregnancy [39].

Recent treatment options

Ibuprofen, naproxen sodium, diclofenac potassium, and acetylsalicylic acid, along with the seven triptans sumatriptan, rizatriptan, eletriptan, zolmitriptan, almotriptan, frovatriptan, and naratriptan, are acceptable medications for the treatment of acute migraines, according to recent clinical trials, meta-analyses, and practice guidelines [40]. New therapies, such as the calcitonin gene-related peptide monoclonal antibodies, lasmiditan, direct calcitonin gene-related peptide antagonists, and neuromodulation devices, are available for the treatment of headaches [10].

## Conclusions

The prevalence of migraine is more common in females than in males. The understanding and treatment plan for migraine can be understood by understanding the four stages of a migraine, which include premonitory, aura, headache, and postdrome. As its pathogenesis is affected by estrogen, migraine is a neurobiological condition that disproportionately impacts women of reproductive age. The most secure acute migraine treatment during pregnancy is acetaminophen, though acetaminophen with codeine is also a possibility. For some people, sumatriptan during pregnancy may be a choice, and it is safe to take while breastfeeding. Metoclopramide and diphenhydramine together may be a more efficient therapy for migraine or tension headaches than codeine. Triptans might not have any negative impacts on the fetus or child. Low-dose aspirin may not be linked to fetal/child deleterious effects, but acetaminophen, prednisolone, indomethacin, ondansetron, antipsychotics, and parenteral magnesium may be. Our research indicates that women are more likely than men to experience migraine attacks, but this tendency decreases during pregnancy and lactation due to steady estrogen levels. Acetaminophen, ergot alkaloids, NSAIDs, and triptan can all be used to manage migraines. Non-pharmacological treatment should be regarded as first-line therapy before beginning drug therapy. Acetaminophen should be regarded as harmless for the short-term therapy of migraines during pregnancy. However, it can result in HKD and ADHD. Aspirin at low doses is also thought to be harmless for treating migraines during pregnancy. Triptan should be used for intense migraine attacks.
